# Two *Cyc2CL* transcripts (*Cyc2CL-1* and *Cyc2CL-2*) may play key roles in the petal and stamen development of ray florets in chrysanthemum

**DOI:** 10.1186/s12870-021-02884-z

**Published:** 2021-02-19

**Authors:** Hua Liu, Ming Sun, Huitang Pan, Tangren Cheng, Jia Wang, Qixiang Zhang

**Affiliations:** 1 Beijing Key Laboratory of Ornamental Plants Germplasm Innovation & Molecular Breeding, National Engineering Research Center for Floriculture, Beijing Laboratory of Urban and Rural Ecological Environment, Engineering Research Center of Landscape Environment of Ministry of Education, Key Laboratory of Genetics and Breeding in Forest Trees and Ornamental Plants of Ministry of Education, School of Landscape Architecture, Beijing Forestry University, Beijing, 100083 China; 2 Beijing Advanced Innovation Center for Tree Breeding by Molecular Design, Beijing Forestry University, Beijing, 100083 China

**Keywords:** *Chrysanthemum morifolium*, Ray florets, Disc florets, *CYC2*-like genes, Stamen abortion, Alternative splicing

## Abstract

**Background:**

*Chrysanthemum morifolium* is one of the most popular ornamental crops. The capitulum, which is the main ornamental part of chrysanthemum plants, consists of ligulate marginal ray florets, an attractive corolla (petals), and radially hermaphroditic disc florets, but no stamens. In Asteraceae species, the zygomorphic ray florets evolved from the actinomorphic disc florets. During this process, the zygomorphic ligulate corolla arose and the stamens were aborted. Although molecular genetic research has clarified ray floret development to some extent, the precise molecular mechanism underlying ray floret development in chrysanthemum remained unclear.

**Results:**

A *CYC2*-like gene, *Cyc2CL*, was cloned from *C. morifolium* ‘Fenditan’. Subsequent analyses revealed that the alternative splicing of *Cyc2CL*, which occurred in the flower differentiation stage, resulted in the production of *Cyc2CL-1* and *Cyc2CL-2* in the apical buds. Prior to this stage, only *Cyc2CL-1* was produced in the apical buds. A fluorescence in situ hybridization analysis of labeled *Cyc2CL-1* and *Cyc2CL-2* RNA indicated that *Cyc2CL-2* was first expressed in the involucre tissue during the final involucre differentiation stage, but was subsequently expressed in the receptacle and floret primordia as the floral bud differentiation stage progressed. Moreover, *Cyc2CL-2* was highly expressed in the inflorescence tissue during the corolla formation stage, and the expression remained high until the end of the floral bud differentiation stage. Furthermore, the overexpression of *Cyc2CL-1* and *Cyc2CL-2* in transgenic Arabidopsis inhibited stamen and petal development. Therefore, both *Cyc2CL-1* and *Cyc2CL-2* encode candidate regulators of petal development and stamen abortion and are important for the ray floret development in chrysanthemum.

**Conclusion:**

In this study, we characterized the alternatively spliced transcripts of the *CYC2*-like gene that differ subtly regarding expression and function. The data presented herein will be useful for clarifying the regulatory mechanisms associated with the *CYC2*-like gene and may also be important for identifying the key genes and molecular mechanisms controlling the development of ray florets in chrysanthemum.

**Supplementary Information:**

The online version contains supplementary material available at 10.1186/s12870-021-02884-z.

## Background

Asteraceae is the largest family of flowering plants, and it belongs to the euasterids II clade of the core eudicots. The unique head-like inflorescence of Asteraceae species, known as a capitulum, often consists of the following two morphologically and functionally differentiated florets: bilateral (zygomorphic) ray florets and radial (actinomorphic) disc florets [18]. The marginal ray florets are ligulate and female, with an attractive corolla (petals), but no stamens. The inner disc florets with fertile pollen grains are radially pentamerous and hermaphroditic. In Asteraceae species, zygomorphic ray florets evolved from the actinomorphic disc florets [41]. In angiosperms, the transition to bilateral floral symmetry is considered to represent one of three critical evolutionary events, and is related to the evolution of specialized flower–pollinator interactions that contributed to the diversification of flowering plant lineages [1, 12, 20].

The development of the zygomorphic female ray floret was an important event associated with the evolutionary success of the capitulum in Asteraceae species because the ray floret is highly attractive to pollinators and can significantly improve the outcrossing rate [44]. The following two fundamental changes occurred during the development of the zygomorphic female ray floret: the zygomorphic ligulate corolla arose and the stamens were aborted. Many recent phylogenetic and biological studies have focused on this trait.

In *Antirrhinum majus* (Lamiales), the TCP transcription factors CYCLOIDEA (CYC) and DICHOTOMA (DICH) were isolated and characterized as key regulators of floral zygomorphy [33]. Several *CYC*-like TCP-encoding genes have been identified in Asteraceae species, and the *CYCLOIDEA*/*TEOSINTE BRANCHED1* (*CYC*/*TB1*)-like subfamily was classified into the following three *CYC* clades: *CYC1*, *CYC2*, and *CYC3*, of which *CYC2* clade genes underwent multiple duplication events, resulting in the functional diversification of these genes within the Asteraceae lineage [43]. The 10 *CYC*/*TB1*-like genes identified in sunflower belong to three distinct clades (*CYC1*, *CYC2*, and *CYC3*), which is consistent with what has been determined for other eudicot species. Previous studies proposed that gene duplication and functional divergence have greatly facilitated the diversification of the sunflower *CYC* gene family [3, 5]. Additionally, a phylogenetic analysis of *CYC*-like genes revealed that different paralogs of these genes may have been independently recruited to mediate the zygomorphy in different Asteraceae species [7]. In *Gerbera hybrida*, the *CYC*-like homolog *GhCYC2* is specifically expressed in the marginal zygomorphic ray florets, but not in the center-most actinomorphic disc flowers. The overexpression of *GhCYC2* results in the production of disc flowers that are morphologically similar to ray flowers. Moreover, GhCYC2 is reportedly important for the differentiation of flower types in *G. hybrida* [2]. In *Senecio* species, RAY1 is important for regulating floret identity (i.e., ray or disc florets), whereas RAY2 promotes the ventral identity of ray florets. The *RAY1* and *RAY2* genes belong to a subfamily of *TCP* genes, and *RAY2* may be an ortholog of *GhCYC2* in *G. hybrida* [34]. In a previous study of sunflower mutants, a *CYC*-like gene, *HaCYC2c*, was expressed throughout the inflorescence, and disc florets developed bilateral symmetry [7]. Therefore, in Asteraceae species, some *CYC2* clade genes that are specifically expressed in ray florets determine floret identity (i.e., ray or disc florets). Examples include *GhCYC2* in *G. hybrida*, *HaCYC2d* and *HaCYC2c* in sunflower, and *RAY1* and *RAY2* in *Senecio vulgaris*.

In an earlier investigation, tissue slices were analyzed to study early stamen development and the subsequent abortion in *Gerbera* species. The results indicated that in the early stages, the stamen primordia in ray and disc florets develop similarly, but the development of the stamen primordium in ray florets subsequently starts to lag behind the corresponding development in disc florets. Additionally, the stamens of ray florets are gradually aborted [29]. However, little is known about the molecular mechanisms responsible for stamen abortion in ray florets.

The alternative splicing (AS) of precursor mRNAs (pre-mRNAs) enables the same gene to generate multiple transcripts that may encode various protein isoforms. Alternative splicing has profound functional consequences because of the resulting changes to protein production. Moreover, AS is broadly useful for enhancing molecular versatility. Specifically, it is a key gene regulatory process that influences almost all analyzed biological functions. Additionally, AS increases the coding potential of genomes, and represents an important post-transcriptional regulatory mechanism for increasing the proteomic diversity and functional complexity of higher eukaryotes. Furthermore, AS is common in plants [4, 39]. However, whether *CYC2*-like genes are affected by AS remains unknown.

*Chrysanthemum morifolium*, which is one of the most popular ornamental crops, is cultivated worldwide [19, 40]. The main ornamental part of *C. morifolium* plants is the capitulum, and its typical structure contains morphologically distinct ray and disc florets (Supplemental Figure S1). Ray florets, which are ligulate and zygomorphic, have a showy corolla (petals) and lack stamens. Their primary function is to attract pollinators. The central disc florets, which are radially symmetrical and hermaphroditic, have fertile pollen grains and are mainly required for reproduction [31]. In this study, a *CYC2*-like gene, *Cyc2CL*, was cloned from *C. morifolium* ‘Fenditan’. The AS of *Cyc2CL* resulted in two distinct transcripts (*Cyc2CL-1* and *Cyc2CL-2*), which were produced in the apical buds after the flower differentiation stage was initiated. Prior to this stage, only *Cyc2CL-1* was produced in apical buds. Additionally, *Cyc2CL-2* was first expressed in the involucre tissue during the final involucre differentiation stage. As the floral bud differentiation stage proceeded, *Cyc2CL-2* was expressed in the receptacle and floret primordia. Studies involving transgenic Arabidopsis plants revealed that the overexpression of *Cyc2CL-1* and *Cyc2CL-2* can inhibit the development of stamens and petals. Therefore, Cyc2CL may play a key role in controlling the stamen abortion and petal development of ray florets and is likely a crucial regulator of chrysanthemum ray floret development. The results of this study are important for clarifying the molecular mechanisms underlying ray floret development in chrysanthemum and may be useful for identifying important candidate genes for the breeding of chrysanthemum and related species.

## Results

### Isolation of chrysanthemum *CYC* homologs

**Fig. 1:**
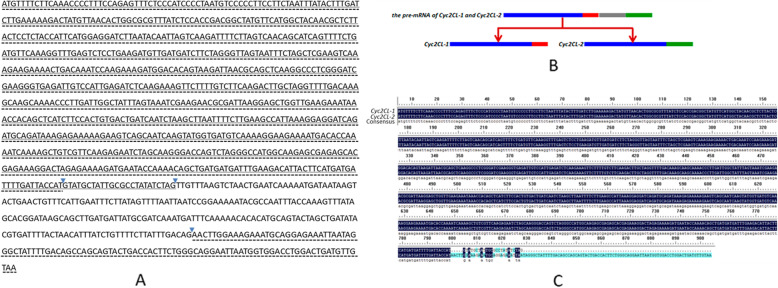
Alternative splicing of *Cyc2CL* in *C. morifolium*. **a** *Cyc2CL-1* and *Cyc2CL-2* pre-mRNA sequences. The *Cyc2CL-1* and *Cyc2CL-2* exons are indicated with solid and dashed lines, respectively. Triangles indicate the splice sites. **b** Alternative splicing of *Cyc2CL* in *C. morifolium*. The blue bar indicates the common exon sequences of *Cyc2CL-1* and *Cyc2CL-2*. The red and green bars indicate the exon sequences of *Cyc2CL-1* and *Cyc2CL-2*, respectively. The gray bar indicates the intron sequences. **c** Alignment of the *Cyc2CL-1* and *Cyc2CL-2* mRNA sequences

**Fig. 2:**
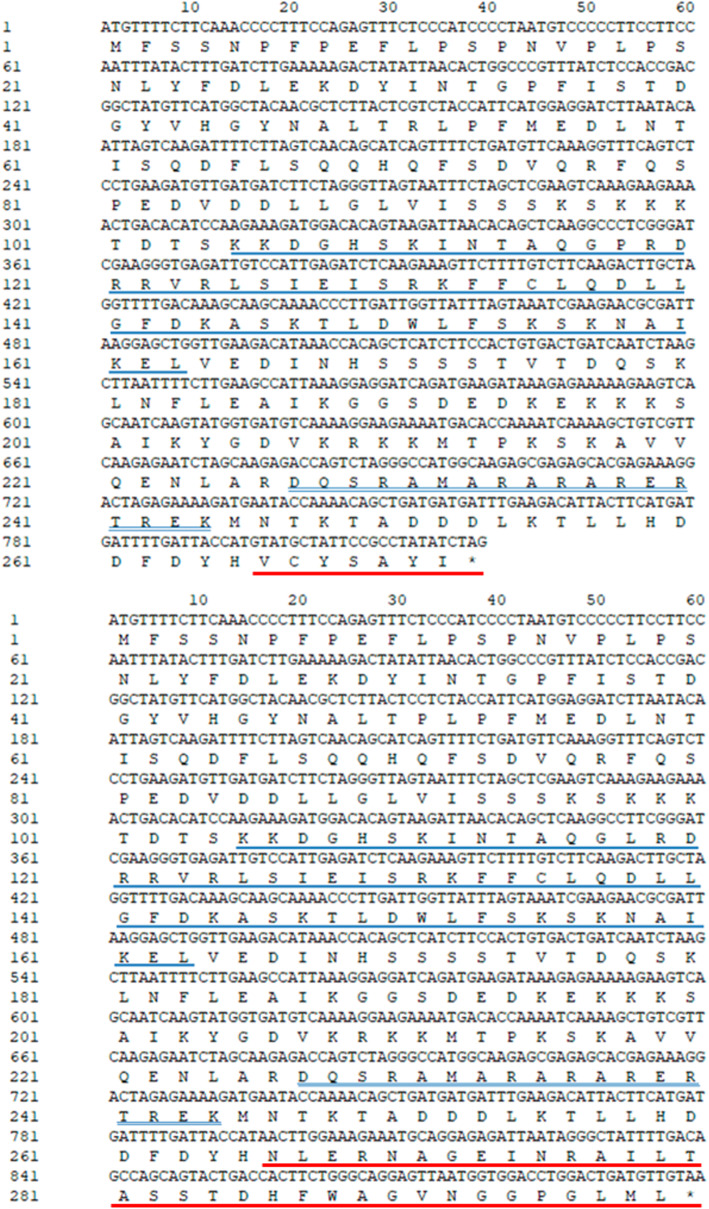
Coding sequences and deduced amino acid sequences of *Cyc2CL-1* and *Cyc2CL-2*. The blue single and double underlines indicate the TCP and R domains, respectively. The differences in the alternatively spliced transcripts are underlined in red

**Fig. 3:**
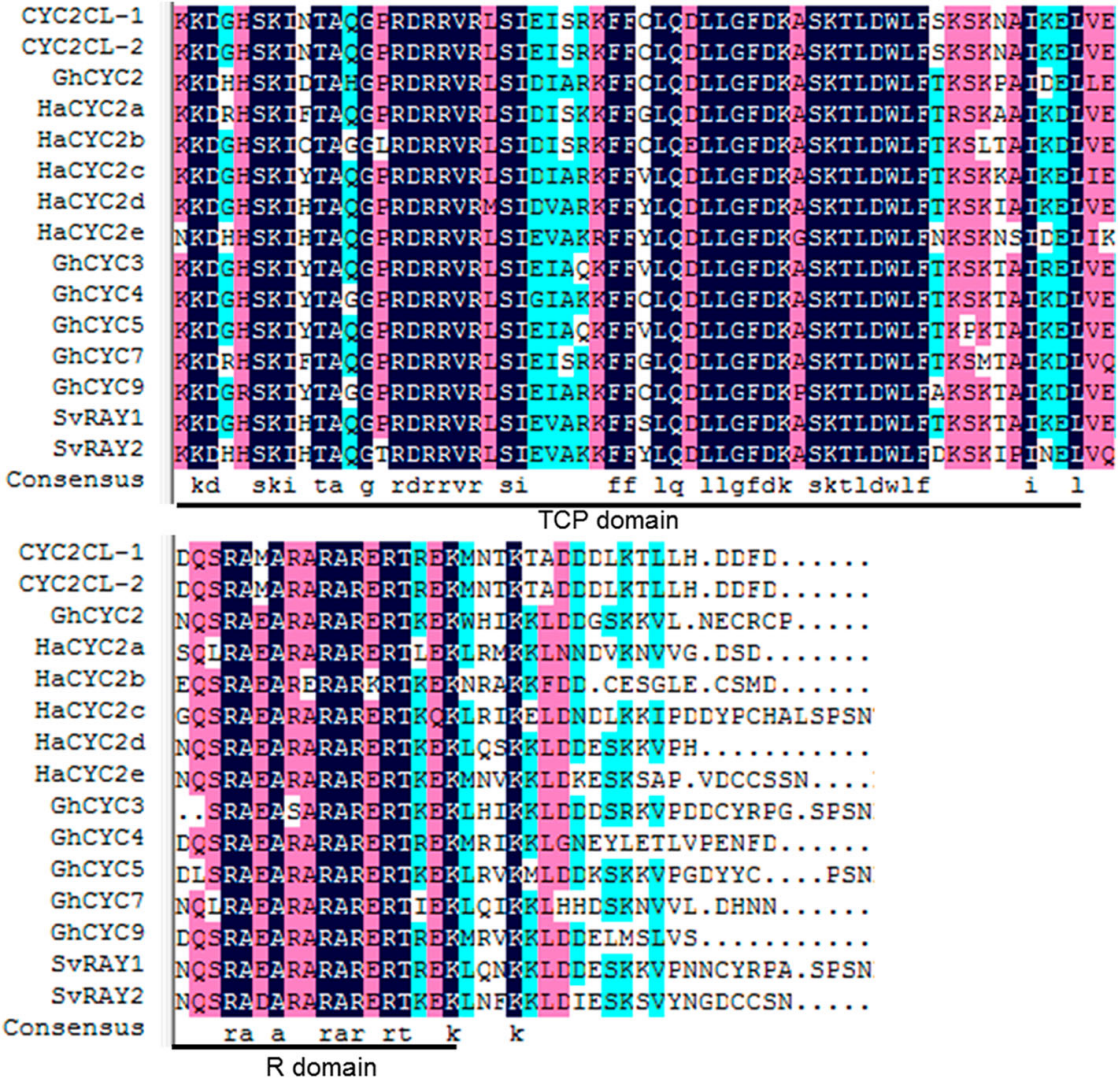
Multiple sequence alignment of CYC proteins from *C. morifolium* and other species. The TCP and R domains are indicated by lines below the aligned sequences. The Genbank accession numbers for the genes in the sequence alignment are as follows: *CYC2CL-1* (*Chrysanthemum morifolium*, AIU94285.1), *CYC2CL-1* (*C. morifolium*, BAC11907.1), *GhCYC2* (*Gerbera hybrida*, ACC54347.1), *HaCYC2a* (*Helianthus annuus*, ABV26442.1), *HaCYC2b* (*H. annuus*, ABV26443.1), *HaCYC2c* (*H. annuus*, ABV26444.1), *HaCYC2d* (*H. annuus*, ABV26445.1), *HaCYC2e* (*H. annuus*, ABV26446.1), *GhCYC3* (*G. hybrida*, ACC54348.1), *GhCYC4* (*G. hybrida*, ACC54349.1), *GhCYC5* (*G. hybrida*, AEX07362.1), *GhCYC7* (*G. hybrida*, AEX07364.1), *GhCYC9* (*G. hybrida*, AEX07366.1), *SvRAY1* (*Senecio vulgaris*, ACJ71723.1), and *SvRAY2* (*S. vulgaris*, ACJ71727.1)

**Fig. 4:**
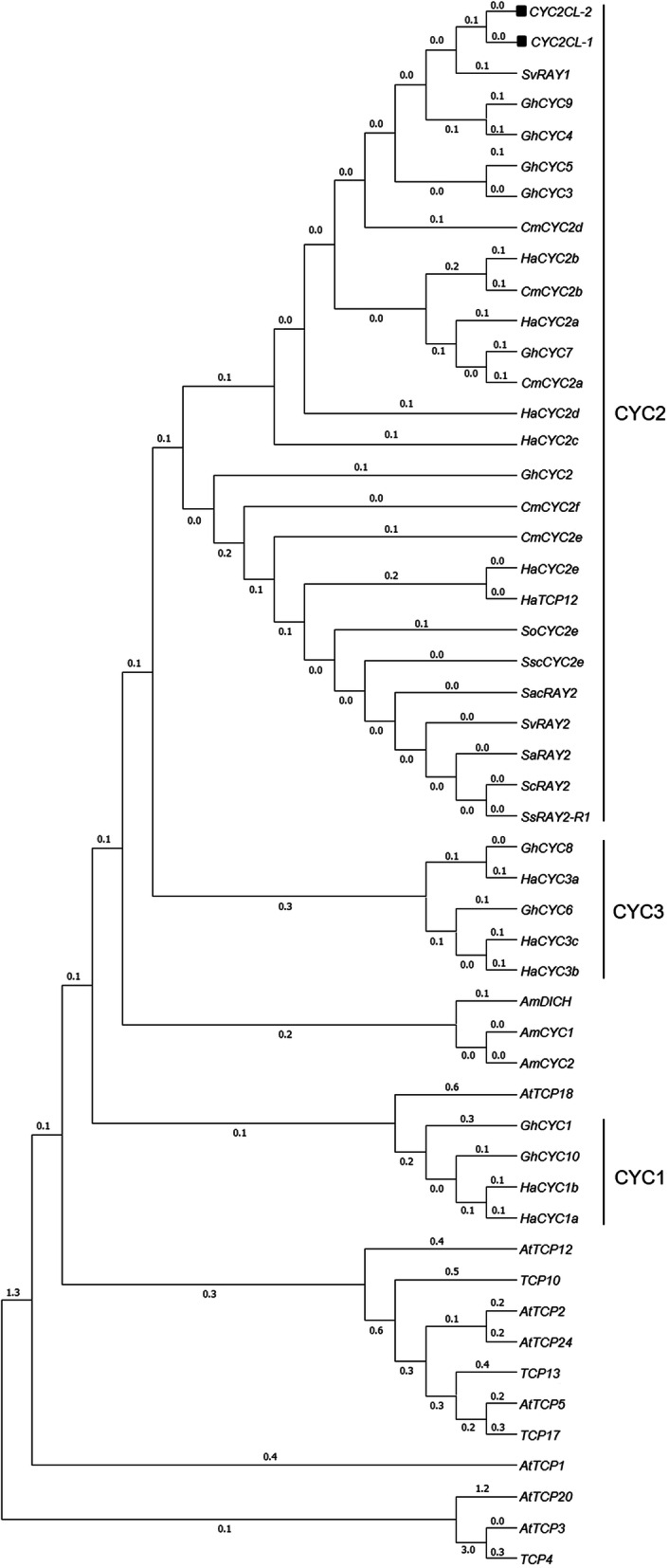
Phylogenetic tree of *CYC* genes from *C. morifolium* and other species. The Genbank accession numbers for the genes used to construct the phylogenetic tree are as follows: *GhCYC5*: AEX07362.1, *HaCYC2c*: ABV26444.1, *GhCYC3*: ACC54348.1, *GhCYC7*: AEX07364.1, *HaCYC2a*: ABV26442.1, *SvRAY1*: ACJ71723.1, *HaCYC2d*: ABV26445.1, *GhCYC9*: AEX07366.1, *GhCYC4*: ACC54349.1, *GhCYC2*: ACC54347.1, *SvRAY2*: ACJ71727.1, *HaCYC2e*: ABV26446.1, *HaCYC2b*: ABV26443.1, *CYC2CL-1*: CAD23438.1, *CYC2CL-2*: AAK21248.1, *CmCYC2a*: KU595430.1, *CmCYC2b*: KU595431.1, *CmCYC2d*: KU595426.1, *CmCYC2e*: KU595427.1, *CmCYC2f*: KU595429.1, *GhCYC10*: AEX07367.1, *GhCYC1*: ACC54346.1, *HaCYC1b*: ABV26441.1, *HaCYC1a*: ABV26440.1, *HaCYC3a*: ABV26447.1, *GhCYC8*: AEX07365.1, *GhCYC6*: AEF59025.1, *GhCYC8*: AEX07365.1, *GhCYC6*: AEX07363.1, *HaCYC3c*: ABV26449.1, *HaCYC3b*: ABV26448.1, *AmDICH*: AAF12817.1, *AmCYC1*: Q9SBV9.1, *AmCYC2*: O49250.1, *AtTCP3*: AEE32909.1, *AtTCP5*: AED97405.1, *AtTCP2*: AEE84040.1, *AtTCP24*: AEE31193.1, *AtTCP20*: AEE77254.1, *AtTCP18*: OAP04988.1, *AtTCP1*: OAP12772.1, *AtTCP12*: AEE34841.1, *AtTCP4*: EU550941.1, *AtTCP10*: EU550953.1, *AtTCP13*: XP_020886906.1, *AtTCP17*: NM_001342977, *SscCYC2e*: *MG593448*.*1*, *SoCYC2e*: MG593440.1, *ScRAY2*:JQ351921.1, *SacRAY2*: JQ351929.1, *SaRAY2*: JQ351911.1, *SsRAY2-R1*: FJ356704.1, and *HaTCP12*: XP_022034966.1

We isolated the pre-mRNA sequences (1146 bp) and the two alternatively spliced transcripts (*Cyc2CL-1* and *Cyc2CL-2*) of the *CYC*-like gene *Cyc2CL* (Fig. 1). The full-length *Cyc2CL-1* and *Cyc2CL-2* genomic sequences were also cloned (Supplemental Figure S2). As shown in Fig. 1b, the splice variants are derived from a pre-mRNA transcript and alternative splicing produces two types of mRNA. The *Cyc2CL-1* exon sequences contained 819 bp. Regarding *Cyc2CL-2*, the first exon comprised 795 bp and was similar to the *Cyc2CL-1* exon, whereas the second exon consisted of 105 bp, which matched part of the *Cyc2CL-1* intron. The *Cyc2CL-2* exons were separated by an intron sequence (246 bp). Thus, *Cyc2CL-1* and *Cyc2CL-2* shared partly similar exon sequences, but had diverse intron sequences. The splice sites of the *Cyc2CL-2* exons and intron are consistent with the canonical GT-AG splice sites, whereas the splice sites of the *Cyc2CL-1* exon and intron are non-canonical sequences [38]. The *Cyc2CL-1* and *Cyc2CL-2* coding sequences were 819 and 900 bp, respectively. The encoded Cyc2CL-1 and Cyc2CL-2 amino acid sequences were 89% similar (Fig. 1c). The deduced Cyc2CL-1 and Cyc2CL-2 amino acid sequences included the conserved TCP and R domains typical of CYC/TB1 subfamily members (Figs. 2 and 3). During phylogenetic analyses, *Cyc2CL-1* and *Cyc2CL-2* were clustered with the other *CYC2* members in *C. morifolium* as well as with the *CYC2*-like genes of *Helianthus annuus*, *G. hybrida*, and *S. vulgaris*, implying there may have been several *CYC2* subclade gene duplication events in Asteraceae species (Fig. 4).

### Expression analyses of chrysanthemum *CYC* homologs

**Fig. 5:**
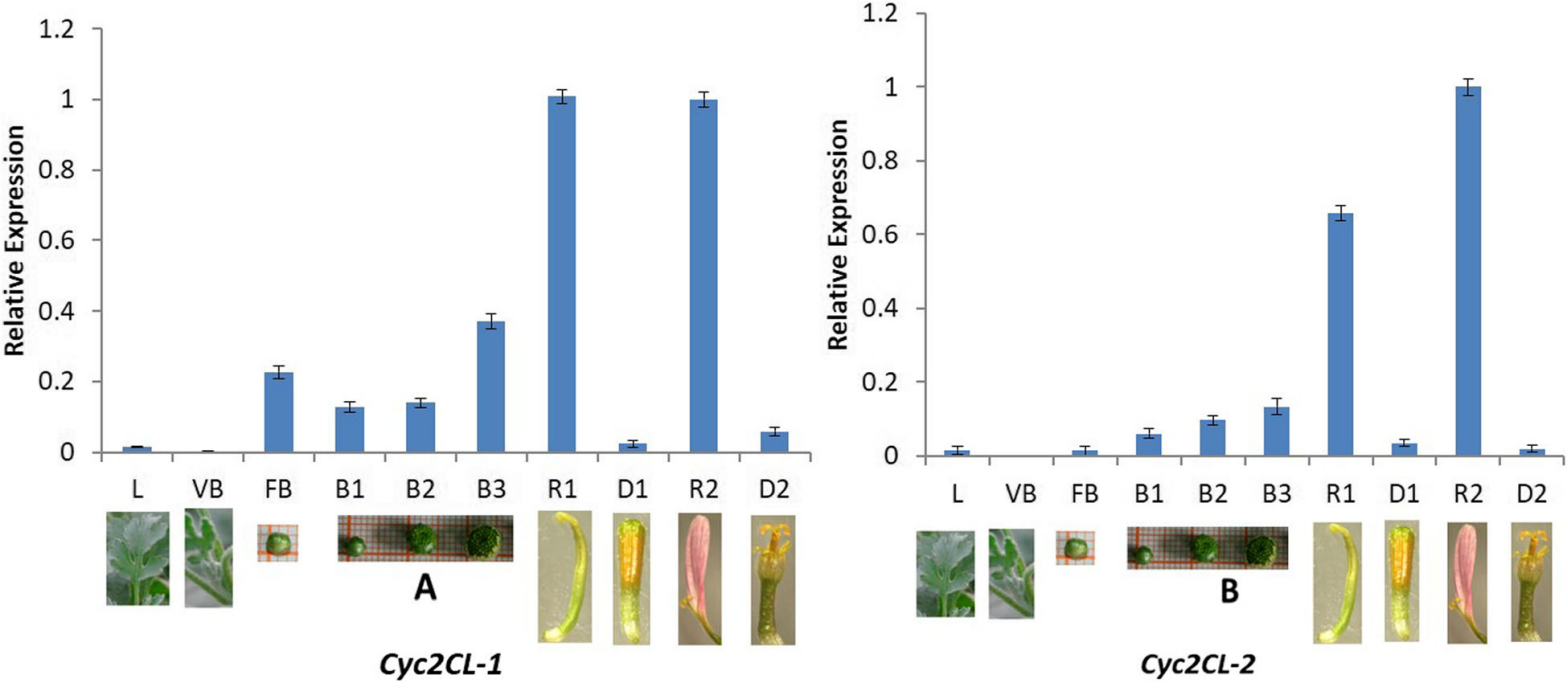
Expression patterns of the *C. morifolium CYC* subfamily genes during various flower developmental stages. L: leaf, VB: vegetative bud, FB: flower bud, B1–B3: buds of three flower developmental stages, R1: ray floret during the early flowering stage, D1: disc floret during the early flowering stage, R2: ray floret during the full flowering stage, and D2: disc floret during the full flowering stage

**Fig. 6:**
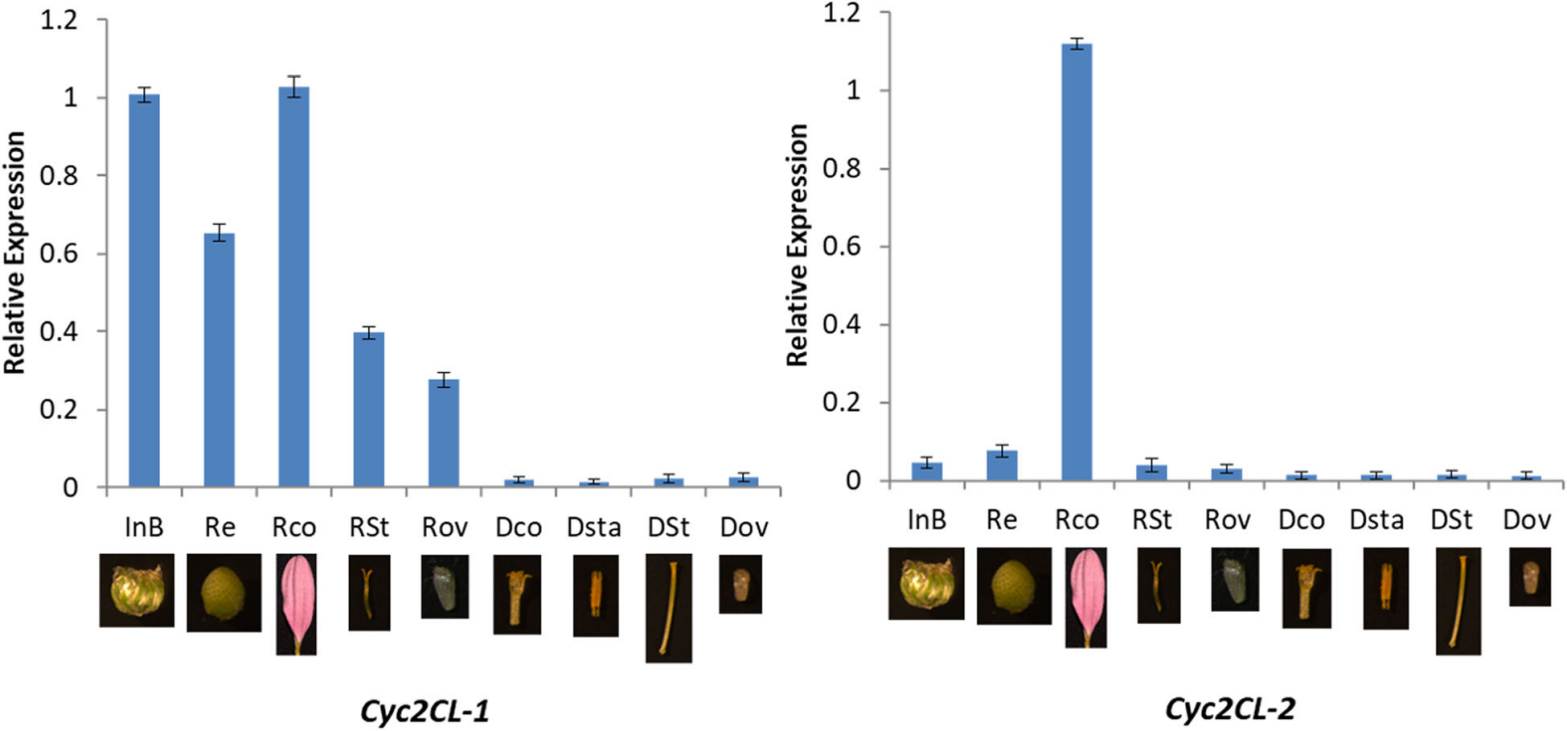
Expression patterns of the *C. morifolium CYC* subfamily genes in different floral tissues. InB: involucral bract, Re: receptacle, RCo: ray floret corolla, RSt: ray floret stigma and style, ROv: ray floret ovary, DCo: disc floret corolla, DSta: disc floret stamen, DSt: disc floret stigma and style, and DOv: disc floret ovary

A quantitative real-time polymerase chain reaction (qRT-PCR) assay was conducted to analyze the expression levels of *CYC*-like genes in chrysanthemum floral parts. Relatively low *Cyc2CL-1* expression levels were detected in the leaves and vegetative buds, whereas *Cyc2CL-2* expression was undetectable in the vegetative buds (Fig. 5). During chrysanthemum bud development, the *Cyc2CL-1* and *Cyc2CL-2* expression levels tended to increase. Additionally, *Cyc2CL-1* and *Cyc2CL-2* were highly expressed in ray florets, but were expressed at low levels in disc florets. These results implied that *Cyc2CL-2* is not expressed in the vegetative buds, but is expressed when floral bud differentiation is initiated. An analysis of gene expression patterns in various floral tissues revealed that *Cyc2CL-1* was highly expressed in the ray floret corolla, involucral bract, and receptacle, and relatively highly expressed in the pistil (stigma, style, and ovary) (Fig. 6). In contrast, *Cyc2CL-2* was mainly expressed in the ray floret corolla. Both *Cyc2CL-1* and *Cyc2CL-2* were expressed at extremely low levels in the disc floret tissues, including the corolla, stamen, and pistil (stigma, style, and ovary) (Fig. 6). Thus, both *Cyc2CL-1* and *Cyc2CL-2* were mainly expressed in floral reproductive organs and weakly expressed in vegetative organs. This is consistent with the results of an earlier investigation on *Gerbera* species and sunflower [2].

**Fig. 7:**
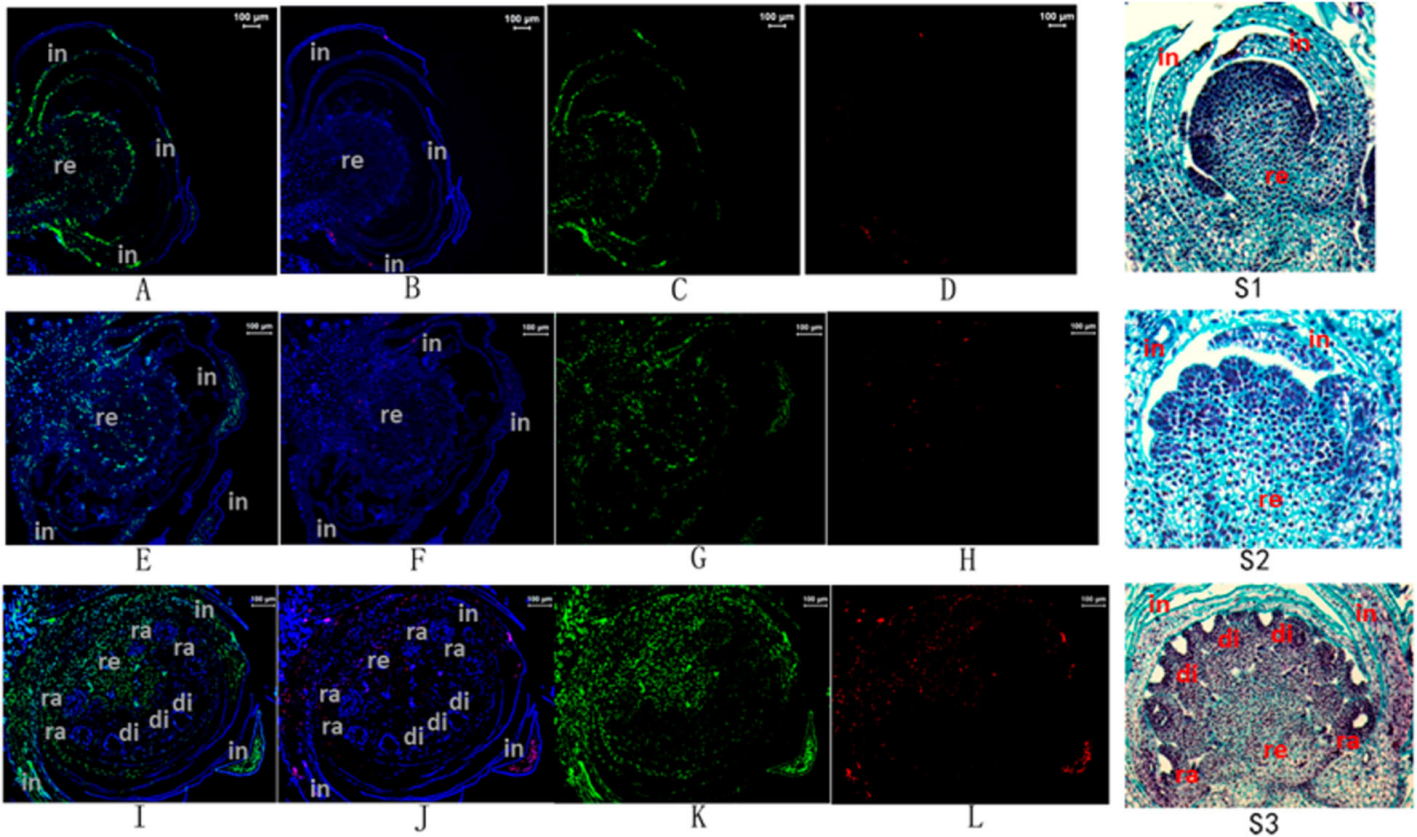
Fluorescence in situ hybridization images of labeled *Cyc2CL-1* and *Cyc2CL-2* RNA in inflorescences. **a**, **c** A FISH image of labeled *Cyc2CL-1* RNA during the final involucre differentiation stage. **b**, **d** A FISH image of labeled *Cyc2CL-2* RNA during the final involucre differentiation stage. **S1**: paraffin section image of the flower bud during the final involucre differentiation stage. **e**, **g** A FISH image of labeled *Cyc2CL-1* RNA during the floret primordia differentiation stage. **f**, **h** A FISH image of labeled *Cyc2CL-2* RNA during the floret primordia differentiation stage. **S2**: paraffin section image of the flower bud during the floret primordia differentiation stage. **i**, **k** A FISH image of labeled *Cyc2CL-1* RNA during the corolla formation stage. **j**, **l** A FISH image of labeled *Cyc2CL-2* RNA during the corolla formation stage. **S3**: paraffin section image of the flower bud during the corolla formation stage. Inflorescence tissues were stained with DAPI (blue). The RNA probes for *Cyc2CL-1* and *Cyc2CL-2* are indicated in green and red, respectively. in: involucre; re: receptacle; ra: ray floret; and di: disc floret

A fluorescence in situ hybridization (FISH) analysis of labeled *Cyc2CL-1* and *Cyc2CL-2* RNA indicated that *Cyc2CL-2* was expressed at low levels in the involucre tissue during the final involucre differentiation stage (Fig. 7b, d). During the floret primordia differentiation stage, *Cyc2CL-2* was expressed in multiple tissues, including the involucre, receptacle, and floret primordia. During the corolla formation stage, which follows the floral bud differentiation stage, *Cyc2CL-2* was highly expressed in the inflorescence tissue. Fluorescence was undetectable in the FISH assay negative controls, in which the sense probes for *Cyc2CL-1* and *Cyc2CL-2* RNA were used (Supplemental Figure 3). Thus, *Cyc2CL-2* was initially expressed in the involucre tissue, but was also expressed in other tissues during the floral bud differentiation stage. The qRT-PCR data confirmed that before the floral bud differentiation stage, *Cyc2CL-2* was not expressed. However, unlike *Cyc2CL-2*, *Cyc2CL-1* was expressed in all inflorescence tissues at relatively high levels.

### Morphological effects of chrysanthemum *CYC* homologs in transgenic Arabidopsis

**Fig. 8:**
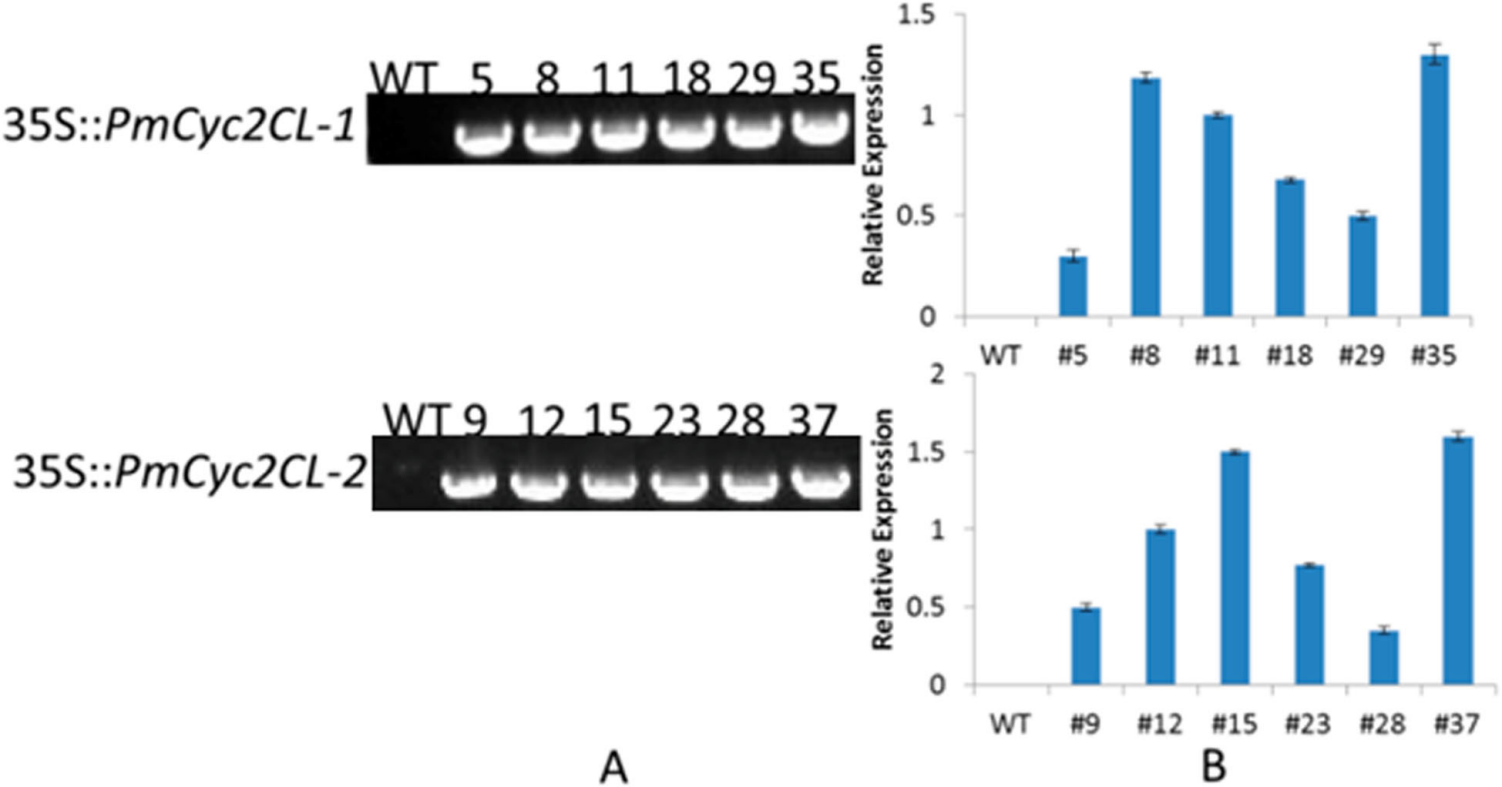
*Cyc2CL-1* and *Cyc2CL-2* expression patterns in transgenic Arabidopsis. **a** Confirmation of gene expression by PCR amplification. **b** Relative gene expression levels as determined in a qRT-PCR assay

**Fig. 9:**
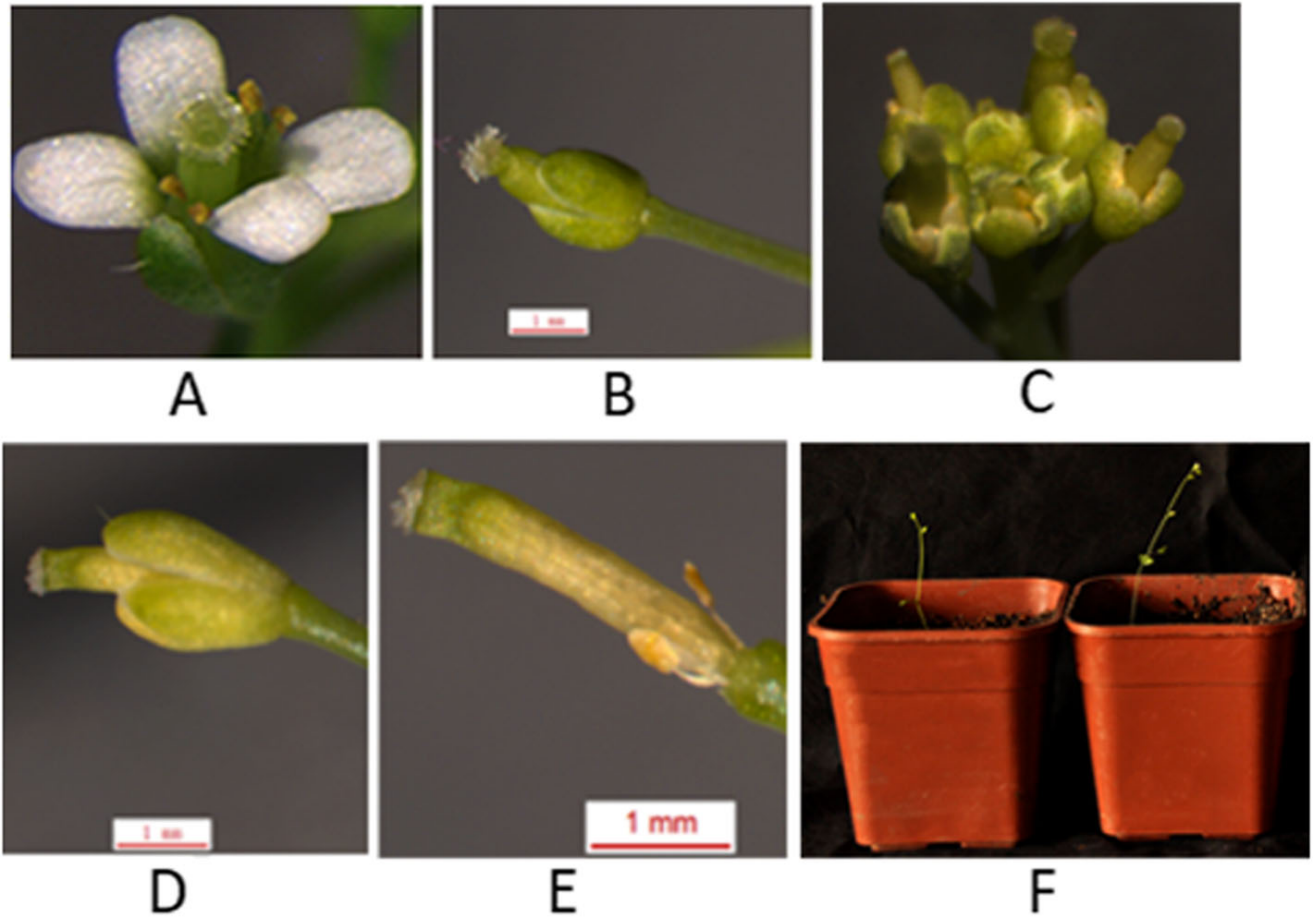
Phenotypes of Arabidopsis plants overexpressing *Cyc2CL-1*. **a** Wild-type flower. **b** Flower of an Arabidopsis plant overexpressing *Cyc2CL-1*. The pistil and sepal grew normally. **c** Inflorescence of an Arabidopsis plant overexpressing *Cyc2CL-1*. The inflorescence had normal pistils and sepals, but the petals and stamens did not grow normally. **d** Flower of an Arabidopsis plant overexpressing *Cyc2CL-1*. Petal and stamen development was suppressed. **e** Flower of an Arabidopsis plant overexpressing *Cyc2CL-1* after sepals were removed. Petals did not develop in this flower. The stigma was normal, but the stamens were aborted. **f** Arabidopsis plants overexpressing *Cyc2CL-1*

**Fig. 10:**
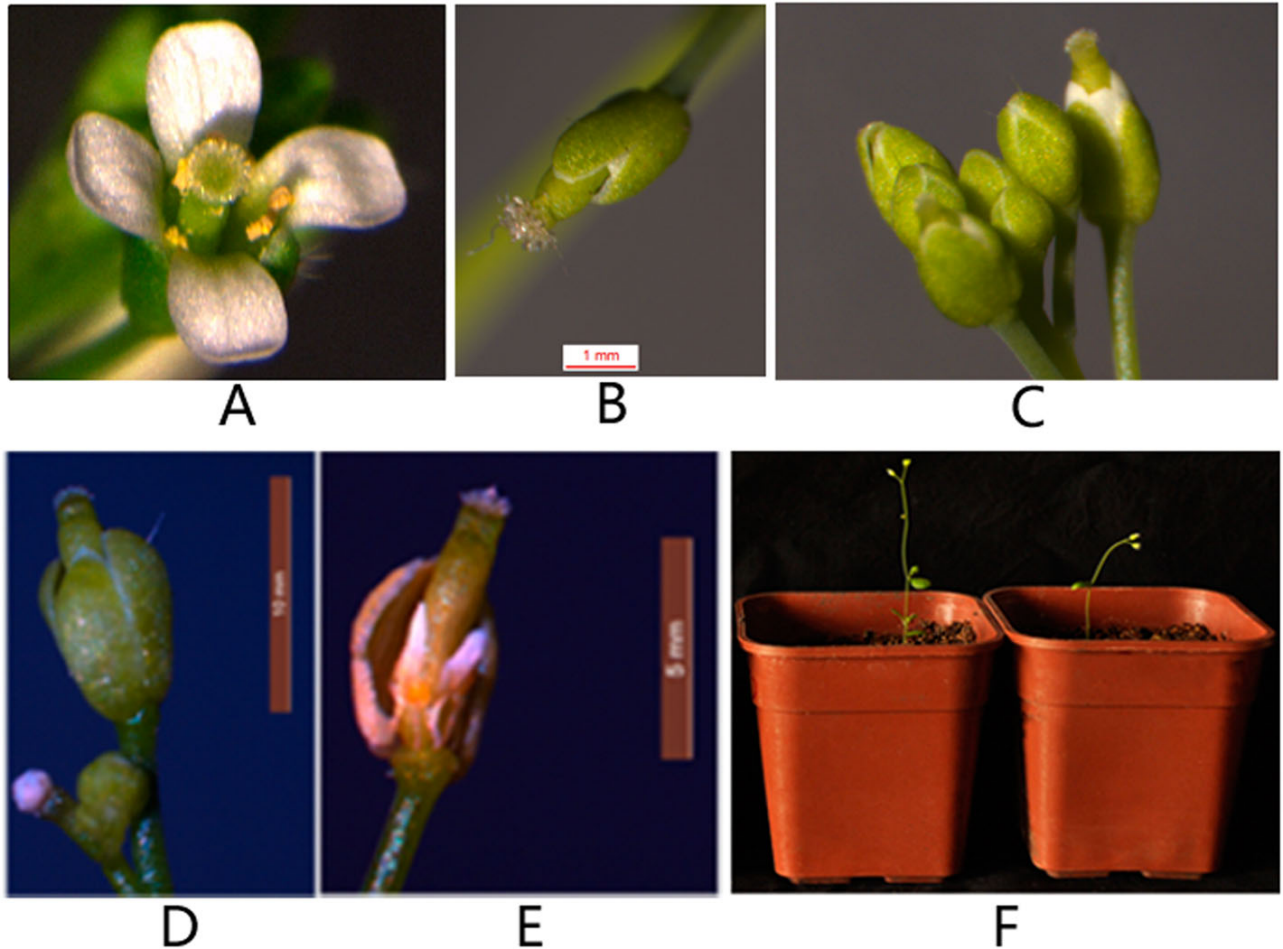
Phenotypes of Arabidopsis plants overexpressing *Cyc2CL-2*. **a** Wild-type flower. **b** Flower of an Arabidopsis plant overexpressing *Cyc2CL-2*. The pistil and sepal grew normally. **c** Inflorescence of an Arabidopsis plant overexpressing *Cyc2CL-2*. The inflorescence had normal pistils and sepals, but the petals and stamens did not grow normally. **d** Flower of an Arabidopsis plant overexpressing *Cyc2CL-2*. Petal and stamen development was suppressed. **e** Flower of an Arabidopsis plant overexpressing *Cyc2CL-2* after the sepals were removed. The petals were abnormally short. The stigma was normal, but the stamens were aborted. **f** Arabidopsis plants overexpressing *Cyc2CL-2*

Two constructs respectively expressing *Cyc2CL-1* and *Cyc2CL-2* under the control of the 35S promoter (35S::*Cyc2CL-1* and 35S::*Cyc2CL-2*) were inserted into Arabidopsis according to the floral-dip method, resulting in 42 and 48 independent lines of 35S::*Cyc2CL-1* and 35S::*Cyc2CL-2* plants, respectively. Most of the transgenic Arabidopsis lines grew weakly compared with the wild-type Arabidopsis plants, and many seedlings died (Supplemental Figure 4). The transgene expression levels of six randomly selected transgenic *Cyc2CL-1*-overexpressing (OE) and *Cyc2CL-2*-OE Arabidopsis lines were analyzed in a qRT-PCR assay (Fig. 8). Of the six *Cyc2CL-1*-OE lines, the highest and lowest *Cyc2CL-1* expression levels were detected in lines #35 and #5, respectively. Among the six *Cyc2CL-2*-OE lines, the highest and lowest *Cyc2CL-2* expression levels were detected in lines #37 and #28, respectively (Fig. 8). The gene expression data confirmed that *Cyc2CL-1* and *Cyc2CL-2* were overexpressed in the transgenic Arabidopsis plants (Figs. 9 and 10). On the basis of *Cyc2CL-1* and *Cyc2CL-2* expression levels, *Cyc2CL-1*-OE Arabidopsis lines #35 and #8 as well as *Cyc2CL-2*-OE Arabidopsis lines #37 and #15 were selected for a subsequent phenotype analysis. In the analyzed *Cyc2CL-1*-OE and *Cyc2CL-2*-OE Arabidopsis lines, the stamens were aborted and the petals were very short or absent, whereas the pistils and sepals were normal. Additionally, wild-type plants had six stamens, but the *Cyc2CL-1*-OE Arabidopsis plants produced only two stamens that were aborted and lacked pollen grains (Fig. 9e). The wild-type Arabidopsis plants had four petals, which was in contrast to the suppressed petal development in the *Cyc2CL-1*-OE Arabidopsis plants. In the examined *Cyc2CL-2*-OE Arabidopsis lines, the petals grew, but were considerably shorter than the wild-type petals, suggesting that petal development was severely inhibited in the *Cyc2CL-2*-OE Arabidopsis lines (Fig. 10e). Similar to the *Cyc2CL-1*-OE Arabidopsis lines, the *Cyc2CL-2*-OE Arabidopsis lines produced two stamens that were aborted and lacked pollen grains. Additionally, normal pistils and sepals were detected in the *Cyc2CL-2*-OE Arabidopsis plants. Therefore, the *Cyc2CL-1*-OE and *Cyc2CL-2*-OE Arabidopsis lines were phenotypically similar. In both lines, the petals and stamens developed abnormally, and the stamens were aborted, resulting in a lack of pollen production. However, the pistils and sepals in the *Cyc2CL-1*-OE and *Cyc2CL-2*-OE Arabidopsis lines developed normally. To functionally characterize *Cyc2CL-1* and *Cyc2CL-2* regarding their inhibitory effects on native *TCP* genes, a qRT-PCR assay was completed to compare the *TCP2*, *TCP3*, *TCP4*, *TCP10*, and *TCP24* expression levels between the wild-type Arabidopsis plants and the transgenic plants that were selected for the subsequent analysis of phenotypes [27]. The results indicated these genes were similarly expressed in the wild-type and transgenic Arabidopsis plants (Supplemental Figure 5). These results implied that Cyc2CL-1 and Cyc2CL-2 are important for stamen and petal development, but have no effect on pistil and sepal development in transgenic Arabidopsis plants.

## Discussion

### Discovery of the alternative splicing of *Cyc2CL* represents an important step toward revealing the subtle molecular mechanism regulating ray floret development in chrysanthemum

#### Genetic control of ray floret development in Asteraceae

Previous studies involving *G. hybrida*, *S. vulgaris*, and *H. annuus* indicated that some *CYC2* clade genes specifically expressed in ray florets determine the production of ray or disc florets. The regulatory mechanism underlying floral symmetry was originally characterized in *A. majus*. Two partially redundant *CYC2* paralogs, *CYC* and *DICH*, determine the dorsal identity of flowers and control the establishment of the zygomorphy (monosymmetry) in petal and stamen whorls [32, 33]. There has recently been considerable research focused on the regulatory functions of *CYC*-like genes affecting the zygomorphic flowers in Asteraceae, Leguminosae, Gesneriaceae, and other plant families.

In Asteraceae, the *CYC*-like genes are included in the *CYC1*, *CYC2*, and *CYC3* subclades. Functional studies have confirmed that *CYC*-like genes are important for controlling organ growth, both as positive and negative regulators involved in cell proliferation and/or expansion [6, 9, 10, 21, 46]. Some studies have confirmed that *CYC* orthologs have a conserved role in controlling petal growth, and changes in their expressed domains determine whether zygomorphic or actinomorphic flowers are produced [2, 7, 22, 33, 43, 45, 48]. In *S. vulgaris*, *H. annuus*, and *G. hybrida*, CYC functions as a floral symmetry regulator, with a key role in determining floret identity (i.e., disc or ray florets) within the capitulum [2, 5, 43]. Duplication events considerably increased the diversity of the *CYC2* clade genes within the Asteraceae lineage. Additionally, six, six, and five *CYC2* clade genes have been identified in *G. hybrida*, *S. vulgaris*, and *H. annuus*, respectively. Some *CYC2*-like genes expressed exclusively in ray florets are essential for the formation of ray florets. For example, in *H. annuus*, both *HaCYC2c* and *HaCYC2d* are specifically expressed in ray florets. In *G. hybrida*, of the *CYC2*-like genes, *GhCYC2*, *GhCYC3*, *GhCYC4*, *GhCYC5*, and *GhCYC9* are expressed specifically in ray/trans florets [2, 5, 24]. Previous investigations revealed that the overexpression of *GhCYC2* in transgenic lines results in disc florets with ray floret features [2, 24, 37]. Similarly, in *S. vulgaris*, two *CYC2* clade genes, *RAY1* and *RAY2*, are also specifically expressed in ray florets, and both genes mediate the development of ray or disc florets [26]. Therefore, previous studies in *G. hybrida*, *S. vulgaris*, and *H. annuus* indicated that some *CYC2* clade genes that are specifically expressed in ray florets determine the production of ray or disc florets. These genes include *GhCYC2* in *G. hybrida*, *HaCYC2d* and *HaCYC2c* in *H. annuus*, and *RAY1* and *RAY2* in *S. vulgaris*.

#### Identification of the *CYC2* clade gene (*Cyc2CL*) and its two transcripts (*Cyc2CL-1* and *Cyc2CL-2*) expressed in ray and disc florets

In this study, one chrysanthemum *CYC2*-like gene (*Cyc2CL*) and its two alternatively spliced transcripts (*Cyc2CL-1* and *Cyc2CL-2*) were revealed. The AS of *Cyc2CL* was initiated during the floral bud differentiation stage in the apical buds. Gene expression analyses in a qRT-PCR assay proved that during the vegetative growth stage, *Cyc2CL-1* is expressed at low levels, whereas *Cyc2CL-2* is not expressed. Thus, *Cyc2CL* is not alternatively spliced during the vegetative growth stage. However, the initiation of the floral bud differentiation stage resulted in up-regulated *Cyc2CL-1* expression levels as well as induced *Cyc2CL-2* expression, but at low levels. During the subsequent bud development stage, *Cyc2CL-1* expression was initially down-regulated, but then gradually increased as the buds developed (Fig. 5). Similarly, the *Cyc2CL-2* expression levels also increased as the buds developed. When the flowers bloomed, *Cyc2CL-1* and *Cyc2CL-2* were most highly expressed in the ray florets, whereas they were weakly expressed in the disc florets. Therefore, at some point during the floral bud differentiation stage, *Cyc2CL* was alternatively spliced to generate *Cyc2CL-1* and *Cyc2CL-2*.

Chrysanthemum floral bud differentiation is believed to comprise the shoot-tip doming stage, the initial and final involucre differentiation stages, the initial and final floret primordia differentiation stages, and the corolla formation stage [30]. In this study, a FISH analysis of labeled *Cyc2CL-1* and *Cyc2CL-2* RNA was conducted to identify the particular developmental stage and tissue in which the AS of *Cyc2CL* was initiated to produce *Cyc2CL-2*. We detected low *Cyc2CL-2* expression levels in the involucre tissue during the final involucre differentiation stage (Fig. 7b, d). In the subsequent floret primordia differentiation stage, *Cyc2CL-2* was expressed in the receptacle and floret primordia in addition to the involucre tissue (Fig. 7f, h). During the corolla formation stage, *Cyc2CL-2* was highly expressed in the inflorescence tissue (Fig. 7j, l). Therefore, we determined that *Cyc2CL-2* is first produced in involucre tissue and then in other tissues during the floral bud differentiation stage.

Our qRT-PCR analysis confirmed that the AS of *Cyc2CL* was initiated in the floral bud differentiation stage, resulting in the production of *Cyc2CL-2*. As the buds developed, the *Cyc2CL-2* expression levels increased. These results were consistent with those of the FISH analysis. Additionally, *Cyc2CL-1* was expressed in all inflorescence tissues and at higher levels than *Cyc2CL-2*. In sunflower, *HaCYC2c* is expressed in the corolla region of ray flowers and in the tubular flower reproductive organs, including the stamen, stigma, style, and ovary [43]. However, unlike the *CYC2* clade genes specifically expressed in *G. hybrida*, *H. annuus*, and *S. vulgaris* ray florets, both *Cyc2CL-1* and *Cyc2CL-2* were weakly expressed in disc floret tissues.

#### Both *Cyc2CL-1* and *Cyc2CL-2* may be important for regulating stamen and petal growth

In chrysanthemum, the outer-layer ray florets vary regarding petal types and the inner-layer disc florets have diverse orientations. Additionally, the arrangement and combination of both floret types determine the daedal capitula types. Moreover, flower head types and petal types develop independently. Therefore, diverse petal types exist in specific types of flower heads. The complexity in the chrysanthemum petal and flower head types is indicative of a characteristic molecular mechanism involving *CYC* clade regulators. Huang et al. [22] cloned six *CYC2* genes from two chrysanthemum cultivars and their F_1_ progenies. They observed that in one *CYC2* gene-overexpressing *Chrysanthemum lavandulifolium* line, some trans-like florets had short petals and abnormal stamens and were morphologically similar to ray florets [22]. Chen et al. [8] analyzed six *CYC2*-like genes in some Asteraceae species and evaluated the effects of *ClCYC2d* overexpression in *C. lavandulifolium*. They proved that the constitutive expression of *ClCYC2d* suppresses corolla growth during ray floret development [8]. Therefore, the *CYC* genes may be functionally diverse and the encoded proteins may interact to regulate the development of chrysanthemum ray florets.

Phenotypic analyses of the *Cyc2CL-1*-OE and *Cyc2CL-2*-OE Arabidopsis lines verified that the stamens were aborted, and the petals were very short or absent, in contrast to the normal pistils and sepals. Alternative splicing can result in the production of multiple mRNAs from a single gene, thereby increasing the proteomic diversity of higher eukaryotes. It is also a vital mechanism for regulating gene expression during the growth and development of higher plants [25, 42]. The data presented herein imply that *Cyc2CL-1* and *Cyc2CL-2* likely play key regulatory roles related to stamen and petal growth.

The observed inhibitory effects of *Cyc2CL-1* and *Cyc2CL-2* expression on stamen and petal growth are consistent with the results of previous studies. In *A. majus*, both *CYC* and *DICH* are expressed in the dorsal domain of the young floral meristem, thereby retarding petal and stamen growth. At a later stage, *CYC* is expressed throughout the dorsal domain to facilitate petal lobe growth and inhibit stamen development [33]. In *Primulina heterotricha*, when flowers are dorsoventrally differentiated during floral development, two *CYC2* clade genes, *CYC1C* and *CYC1D*, are highly expressed in the dorsal petals and the dorsal/lateral stamens. Additionally, the substantial transcription of *CYC1C* in the lateral staminodes at the late development stage is related to the abortion of the dorsal and lateral stamens. Both *CYC1C* and *CYC1D* may regulate the decrease in the dorsal petal size and the abortion of the dorsal/lateral stamens [15, 47]. Fabio et al. confirmed that in *H. annuus*, *TURF* and *CHRY* are crucial for establishing the corolla symmetry of disc and ray flowers, but these genes also influence carpel and stamen development. Additionally, a loss-of-function mutation to the *CYC* gene reportedly leads to hermaphroditic tubular-like ray florets replacing the normal sterile ray florets [13, 14, 16, 17, 35]. Another study indicated that *CYC*-like genes help regulate stamen and carpel differentiation, likely via their association with the genes controlling the cell cycle and flower organ identity [15]. In *G. hybrida*, the ectopic overexpression of *GhCYC2* disrupts stamen development and causes disc flowers to acquire enlarged and markedly fused petals [2].

Nag et al. [36] confirmed that the expression of *TCP4* (i.e., a key target of miR319a) must be appropriately controlled to ensure normal petal and stamen development, with high *TCP4* expression levels disrupting petal and stamen development in Arabidopsis. The ectopic expression of *Cyc2CL-1* and *Cyc2CL-2* similarly inhibits petal and stamen development. Therefore, the CYC and TCP target genes are likely conserved in chrysanthemum and Arabidopsis.

#### The molecular mechanism underlying *Cyc2CL-1* and *Cyc2CL-2* functions remains to be revealed

We identified a *CYC2* clade gene (*Cyc2CL*) and its two transcripts (*Cyc2CL-1* and *Cyc2CL-2*) and assessed the effects of the ectopic expression of this gene in Arabidopsis to functionally characterize *Cyc2CL-1* and *Cyc2CL-2*. However, the *Cyc2CL-1*-OE and *Cyc2CL-2*-OE Arabidopsis lines were phenotypically similar, and the differences in the functions of the two transcripts were not obvious in the transgenic Arabidopsis plants, likely because of the substantial differences in the flower types and molecular mechanisms between chrysanthemums and Arabidopsis. Chrysanthemums have a capitulum consisting of bilateral (zygomorphic) ray florets and radial (actinomorphic) disc florets, whereas Arabidopsis plants produce only one type of radial flowers. Additionally, the gene regulatory mechanism is more complex in chrysanthemums than in Arabidopsis. The regulatory functions of *Cyc2CL-1* and *Cyc2CL-2* cannot be thoroughly analyzed based solely on the phenotypic changes in the transgenic Arabidopsis plants. Thus, we were unable to elucidate the precise molecular mechanism underlying *Cyc2CL-1* and *Cyc2CL-2* functions. Future investigations should examine the consequences of overexpressing and silencing *Cyc2CL-1* and *Cyc2CL-2* in chrysanthemum to clarify the regulatory functions of *Cyc2CL-1* and *Cyc2CL-2* related to ray floret development. Additionally, our qRT-PCR analysis revealed the *Cyc2CL-1* and *Cyc2CL-2* expression levels in the corolla region and reproductive organs of flowers, but the exact floral organ regions in which these genes are expressed should be determined via in situ RNA hybridization in future studies. Analyses of the proteins interacting with Cyc2CL-1 and Cyc2CL-2 as well as the downstream target genes should also be performed to provide new insights into the molecular mechanism controlling ray floret development in chrysanthemum.

## Methods

### Plant materials and RNA extraction

The individual plants used in this study were all derived by tissue culture from a hybrid of chrysanthemum varieties (i.e., *C. morifolium* ‘Fenditan’, which is a ground cover chrysanthemum variety). The plants were developed in our laboratory and were cultivated in a greenhouse at Beijing Forestry University (116.3°E, 40.0°N) under long-day conditions (16-h light/8-h dark) for 180 days and then under short-day conditions (8-h light/16-h dark) at 24 ± 1 °C. Under long-day conditions, approximately 100 vegetative buds were harvested between 9:00–12:00 am. When the plants were first exposed to short-day conditions, about 100 apical buds were harvested between 9:00–12:00 am every week until visible floral buds formed, after which 100 buds were harvested between 9:00–12:00 am every week until the ray florets developed an observable color. Some harvested plant tissues were immediately placed in liquid nitrogen and stored at − 70 °C for a subsequent RNA extraction step. Other plant tissues were treated and underwent a FISH analysis. Total RNA was extracted with the RNeasy Plant Mini Kit (Qiagen, Beijing, China). The quantity and quality of the extracted RNA were determined with the NanoDrop ND2000 spectrophotometer.

### Isolation of *Chrysanthemum morifolium CYC2* genes and construction of phylogenetic trees

Degenerate primers specific for *CYC2* genes (Table 1) were designed based on the *CYC2* homologs in the NCBI database [2, 5]. Full-length cDNA sequences were obtained with the 3′ and 5′ RACE cDNA amplification kits (Takara, Japan). Total DNA was extracted from harvested tissues with the DNAsecure Plant Kit (TianGen, Beijing, China). Total RNA was extracted from chrysanthemum capitula with the RNAprep Pure Kit (for plants) (TianGen, China). The target sequences were amplified by PCR, which was completed in a final reaction volume of 50 μl comprising 2 μl cDNA (40 ng), 0.4 μl Taq DNA polymerase (Promega), 1 μl forward primer (10 μM), 1 μl reverse primer (10 μM), 5 μl 10× PCR buffer (Promega), 1 μl dNTP (10 mM), 3 μl MgCl_2_ (25 mM), and 36.6 μl ddH_2_O. The PCR conditions were as follows: 94 °C for 5 min; 30 cycles of 94 °C for 30 s, 58 °C for 30 s, and 72 °C for 1 min; 72 °C for 10 min. The amplified gene sequences were ligated to the pMD18-T vector and inserted into *Escherichia coli* DH5α cells, after which the accuracy of the inserted fragments was verified by sequencing. The *Cyc2CL-1* and *Cyc2CL-2* coding sequences were deposited in the GenBank database (accession numbers: KP696775.1 and KP696776.1). We obtained *CYC*-like sequences from the NCBI database. The MEGA program (version 10) [28] was used to construct phylogenetic trees based on the maximum likelihood method involving the JTT matrix-based model [23].

**Table 1:** Degenerate primers used for amplifying *C. morifolium CYC* genes

Gene	Forward primer (5′-3′)	Reverse primer (5′-3′)
*CmCyc*	ATGTTTTCYTCAAACCCYTTTC	GTTTTGCTTGCTTTGTCRAAMCCTA

### Gene expression analysis in a qRT-PCR assay

The abundance of two transcripts (*Cyc2CL-1* and *Cyc2CL-2*) in chrysanthemum flowers at different developmental stages was investigated in a qRT-PCR assay, which was completed with the PikoReal Real-Time PCR system (Thermo Fisher Scientific, Germany). To analyze the *Cyc2CL-1* and *Cyc2CL-2* expression patterns in the shoot apices and buds at different developmental stages, we sampled the vegetative buds (i.e., apical buds during the vegetative growth stage), flower buds (transection diameter of about 2 mm), and buds at three flower developmental stages (first stage: bud transection diameter of about 3 mm; second stage: bud transection diameter of about 5 mm; and third stage: bud transection diameter of about 6–7 mm and colors were detectable in the outer ray florets; Fig. 5). To analyze the *Cyc2CL-1* and *Cyc2CL-2* expression patterns in ray and disc florets at different flower developmental stages, we collected ray and disc florets at the early and full flowering stages. In the early stage, colors were detectable in both ray and disc florets. During the full flowering stage, ray and disc florets had bloomed. Furthermore, to analyze the *Cyc2CL-1* and *Cyc2CL-2* expression patterns in different floral tissues, we collected the involucral bract, receptacle, corolla, and pistil (stigma, style, and ovary) of ray florets as well as the corolla, stamen, and pistil (stigma, style, and ovary) of disc florets (Fig. 6). The leaves of wild-type and transgenic Arabidopsis plants were collected to analyze the *CYC2* and *TCP* gene expression levels. We collected three biological replicates for each tissue. The qRT-PCR was completed with SYBR Premix Ex Taq (TaKaRa) and the PikoReal Real-Time PCR system (Thermo Fisher Scientific). Each reaction was prepared in a total volume of 20 μl containing 2 μl first-strand cDNA as the template. The qRT-PCR conditions were as follows: 95 °C for 30 s; 40 cycles of 95 °C for 5 s, the optimal annealing temperature for 30 s, and 60 °C for 30 s. The *C. morifolium* protein phosphatase 2A gene (*PP2Acs*) was used as the reference gene. Details regarding the qRT-PCR primers are provided in Table 2. The qRT-PCR primers specific for Arabidopsis *TCP* genes are listed in Supplemental Table 1. The qRT-PCR assay was completed with three biological replicates, with samples analyzed in triplicate in each replicate.

**Table 2:** Details regarding the qRT-PCR primers specific for *C. morifolium* genes

Gene	Forward primer (5′-3′)	Reverse primer (5′-3′)
*PP2Acs*	ATCAGAACAGGAGGTCAGGG	TAATTTGTATCGGGGCACTT
*Cyc2CL-1*	CCATGGCAAGAGCGAGAGCAC	GATATAGGCGGAATAGCATAC
*Cyc2CL-2*	GCTGATGATGATTTGAAGACA	GTCCAGGTCCACCATTAATTC

### Vector construction and Arabidopsis transformation

Full-length *Cyc2CL-1* and *Cyc2CL-2* cDNA sequences were amplified by a PCR with gene-specific primers (Table 3). The amplicons were inserted into the pGEM-T vector (Promega), after which the accuracy of the inserted fragments was confirmed by sequencing. The *Cyc2CL-1* and *Cyc2CL-2* sequences were then digested with restriction enzymes and subcloned into pCAMBIA1304 and modified pCAMBIA1304 vectors. The resulting plasmids were inserted into *Agrobacterium tumefaciens* strain EHA105 cells, which were then used to transform Arabidopsis (Col-0) cells according to a floral-dip method [11]. The putative transgenic lines were screened on Murashige and Skoog medium containing 50 mg/l hygromycin. The hygromycin-resistant seedlings (T_0_ generation) were transferred to soil after 14 days and grown at 21–23 °C under long-day conditions. The T_3_ generation plants were subsequently analyzed. Col-0 seeds were obtained from the Arabidopsis Biological Resource Center (www.arabidopsis.org).

**Table 3:** Gene-specific primers used for the PCR amplification of full-length *Cyc2CL-1* and *Cyc2CL-2* cDNA sequences

Gene	Forward primer (5′-3′)	Reverse primer (5′-3′)
*Cyc2CL-1*	ACTAGTATGTTTTCTTCAAACC CCTTTCCAG	GGTGACCCAACTAGATATAGGCGGAATAGC
*Cyc2CL-2*	ACTAGTATGTTTTCTTCAAACC CCTTTCCAG	GGTGACCGACTTACAACATCAGTCCAGGTC

### Fluorescence in situ hybridization

The expression of *Cyc2CL-1* and *Cyc2CL-2* in shoot apices and buds at different developmental stages was analyzed by FISH. We designed LNA-based probes to target the mature *Cyc2CL-1* and *Cyc2CL-2* mRNA sequences (Table 4). Sense probes were used as the FISH assay negative controls (Supplemental Table 2). Shoot apices and buds collected at different developmental stages were fixed with a 4% paraformaldehyde solution for 30 min, after which frozen tissue sections were prepared. The fixed slides were washed twice with PBS, treated with proteinase K at 37 °C for 10 min, and then dehydrated in 70, 85, and 100% ethanol for 5 min each. After a denaturing treatment at 78 °C for 5 min, the probes were added to the slides and allowed to hybridize overnight at 42 °C under humid conditions. The slides were washed with 50% formamide/2× SSC at 43 °C and then with 2× SSC at room temperature to eliminate the non-specific and repetitive RNA hybridizations. Finally, the slides were counterstained with DAPI (Sigma) for 10 min and then observed with a Zeiss LSM 700 Meta confocal microscope.

**Table 4:** Gene-specific probe sequences for the fluorescence in situ hybridization of *Cyc2CL-1* and *Cyc2CL-2*

Gene	Probes sequence (5′-3′)
*Cyc2CL-1*	GTAT GCTAT TCCGC CTATA TCTAG
*Cyc2CL-2*	CAGTACTGACCACTTCTGGGCAGGAATTAATGGTGGACCTGGACTGATGTTGTAA

## Supplementary Information

**Additional file 1.**

## Data Availability

The datasets generated and/or analysed during the current study are available in the GenBank repository at NCBI (accession numbers: KP696775.1 and KP696776.1).

## Abbreviation

AS: Alternative splicing
