# Supplementary material for: Two Cyc2CL transcripts (Cyc2CL-1 and Cyc2CL-2) may play key roles in the petal and stamen development of ray florets in chrysanthemum
Source: BMC Plant Biol. 2021 Feb 19;21:105. doi: 10.1186/s12870-021-02884-z (PMC7893774; doi:10.1186/s12870-021-02884-z)
Supplement: Supplementary file 1 — Additional file 1. [file 12870_2021_2884_MOESM1_ESM.zip › 20210202Additional file 1new.docx]

**Additional file 1**

**Two *Cyc2CL* transcripts (*Cyc2CL-1* and *Cyc2CL-2*) may play key roles in the petal and stamen development of ray florets in chrysanthemum**

**Hua Liu^1^, Ming Sun^1^, Huitang Pan^1^, Tangren Cheng^1^, Jia Wang^1^, Qixiang Zhang^1,2^***

*Affiliation*: **^1^** Beijing Key Laboratory of Ornamental Plants Germplasm Innovation & Molecular Breeding, National Engineering Research Center for Floriculture, Beijing Laboratory of Urban and Rural Ecological Environment, Engineering Research Center of Landscape Environment of Ministry of Education, Key Laboratory of Genetics and Breeding in Forest Trees and Ornamental Plants of Ministry of Education, School of Landscape Architecture, Beijing Forestry University, Beijing, 100083, China.

**^2^** Beijing Advanced Innovation Center for Tree Breeding by Molecular Design, Beijing Forestry University, Beijing, 100083, China

******Corresponding author*

Qixiang Zhang: zqxbjfu@126.com;

**Supplemental Fig. S1** Chrysanthemum flowers. (A) Capitulum. (B) Ray and disc florets.

**Supplemental Fig. S2** *Chrysanthemum morifolium Cyc2CL* genomic sequence. The *Cyc2CL-1* exon and intron sequences are indicated with green and red lines, respectively. The *Cyc2CL-2* exon and intron sequences are indicated with blue and purple dashed lines, respectively. Triangles indicate the splice sites. The sequences in bold indicate the location of degenerate primers.

**Supplemental Fig. S3** Fluorescence *in situ* hybridization images of tissue slices hybridized with the sense probes for *Cyc2CL-1* and *Cyc2CL-2* RNA. (A) A FISH image of the sense probes during the final involucre differentiation stage. (B) A FISH image of the sense probes during the corolla formation stage.

**Supplemental Fig. S4** Transgenic Arabidopsis seedlings. (A) Arabidopsis seedlings. (B–F) Plants that died during the seedling stage.

**Supplemental Fig. S5** Relative *TCP* gene expression levels in the wild-type and transgenic *Arabidopsis* plants as determined in a qRT-PCR assay. (A) Relative *TCP2* expression levels. (B) Relative *TCP3* expression levels. (C) Relative *TCP4* expression levels. (D) Relative *TCP10* expression levels. (E) Relative *TCP24* expression levels. #8: transgenic *Arabidopsis* line #8, #35: transgenic *Arabidopsis* line #35, #15: transgenic *Arabidopsis* line #15, #37: transgenic *Arabidopsis* line #37. Ns means the difference is not significant by t-test when compared to WT *Arabidopsis* line, ^ns^*P*＞0.05. Error bars are ±SD values of three biological and three technical replicates.

**Supplemental Fig. S6** The original and full-length gel and blot image in Figure 8.

We confirm that the images used in Figure 8 and 9, Supplementary 1 and 2 were from the experimental photographs and records of Dr. Hua Liu during the study.

Supplemental Table 1. Details regarding the qRT-PCR primers specific for Arabidopsis genes

| **Gene** | **Forward primer** **(5'-3')** | **Reverse primer** **(5'-3')** |
| --- | --- | --- |
| *AtActin* | GGTGTCATGGTTGGTATGGGTC | CCTCTGTGAGTAGAACTGGGTGC |
| *TCP2* | GCATCAGTCATTCCTCGCTAA | TGAGCCTTTACCCTTATGTTCTG |
| *TCP3* | ATCCCTACCATATCCCTCCC | GCCTTGAAACCGTGCTG |
| *TCP4* | CCCCTTCAGTCCAGTTACAG | CGTGCTGGTATGCGAAA |
| *TCP10* | ACAAGAAGAAAGGAGTAAT | TATGAATAAACTGGACTGAAGGG |
| *TCP24* | GGTGGCAAAGATAGACATAGCA | TGATAACGACAACAGCGAACT |

Supplemental Table 2. Sense probe sequences for the negative controls used in the fluorescence *in situ* hybridization of *Cyc2CL-1* and *Cyc2CL-2*

| **Gene** | **Sense probes sequence** **(5'-3')** |  |
| --- | --- | --- |
| *Cyc2CL-1* | CTAGATATAGGCGGAATAGCATAC |  |
| *Cyc2CL-2* | TTACAACATCAGTCCAGGTCCACCATTAATTCCTGCCCAGAAGTGGTCAGTACTG | |
