# Supplementary figures and images for: Two Cyc2CL transcripts (Cyc2CL-1 and Cyc2CL-2) may play key roles in the petal and stamen development of ray florets in chrysanthemum
Source: BMC Plant Biol. 2021 Feb 19;21:105. doi: 10.1186/s12870-021-02884-z (PMC7893774; doi:10.1186/s12870-021-02884-z)

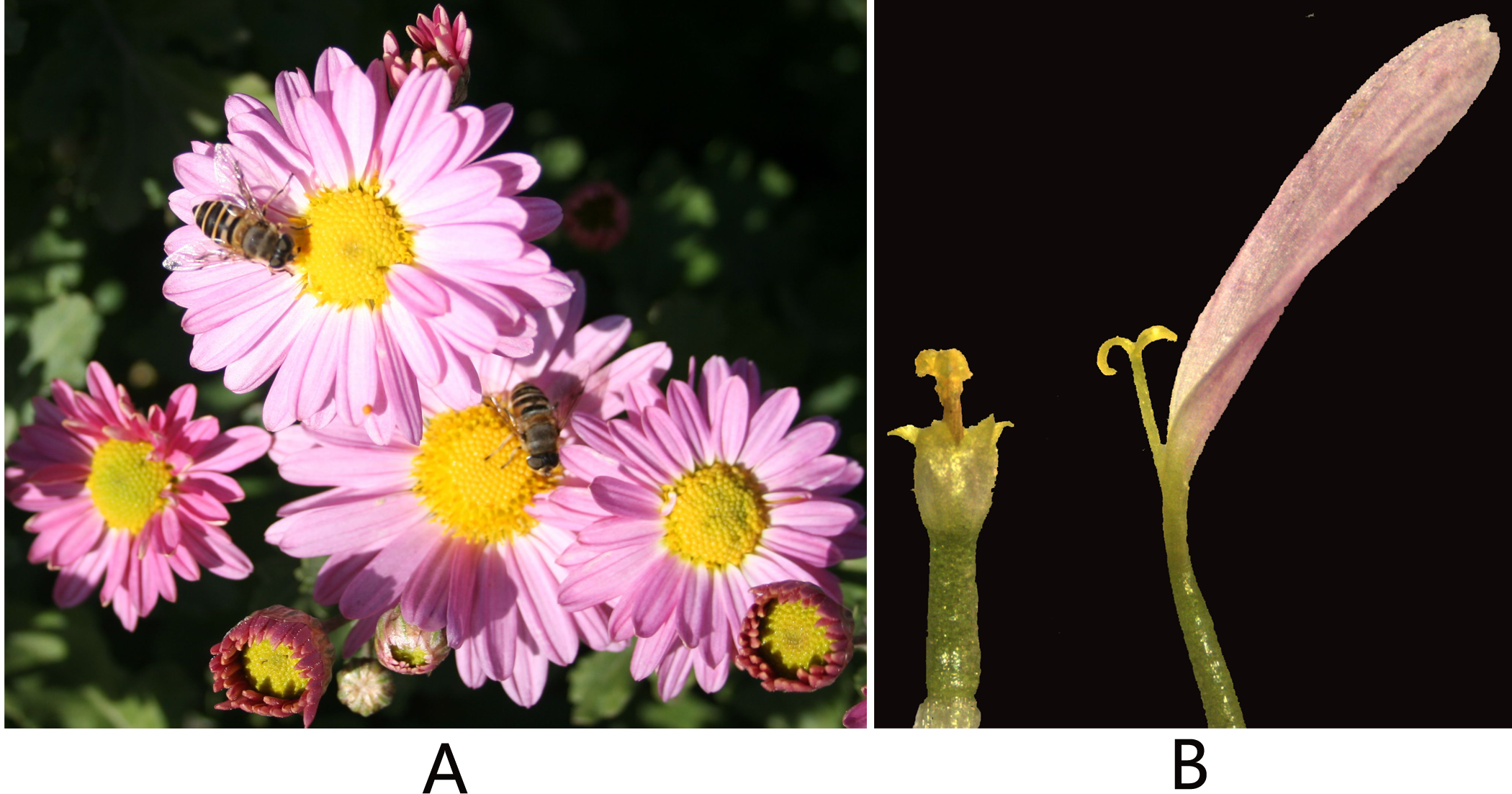

Supplement: Supplementary file 1 — Additional file 1. [file 12870_2021_2884_MOESM1_ESM.zip › Supplemental Fig. 1.tif]

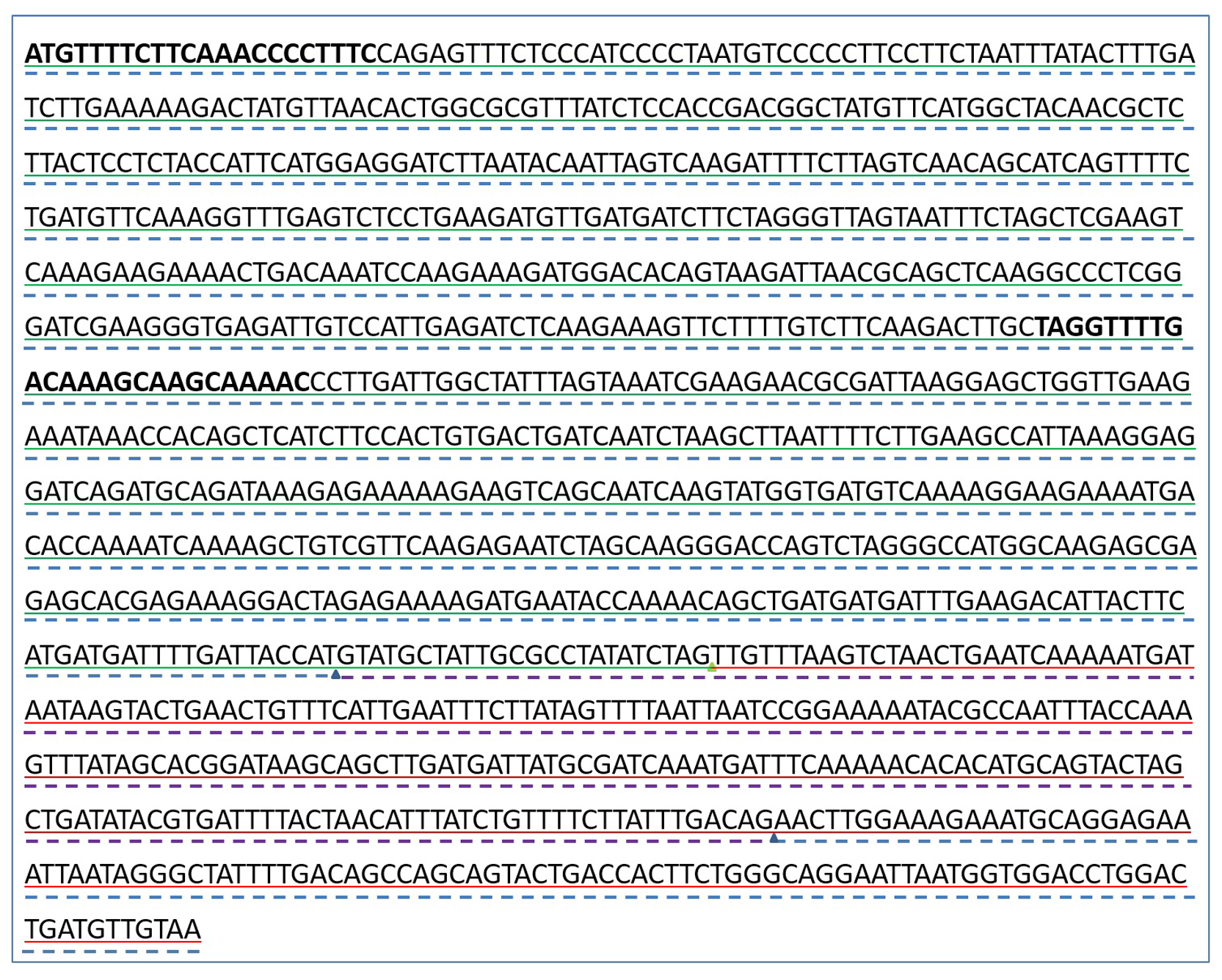

Supplement: Supplementary file 1 — Additional file 1. [file 12870_2021_2884_MOESM1_ESM.zip › Supplemental Fig. 2.tif]

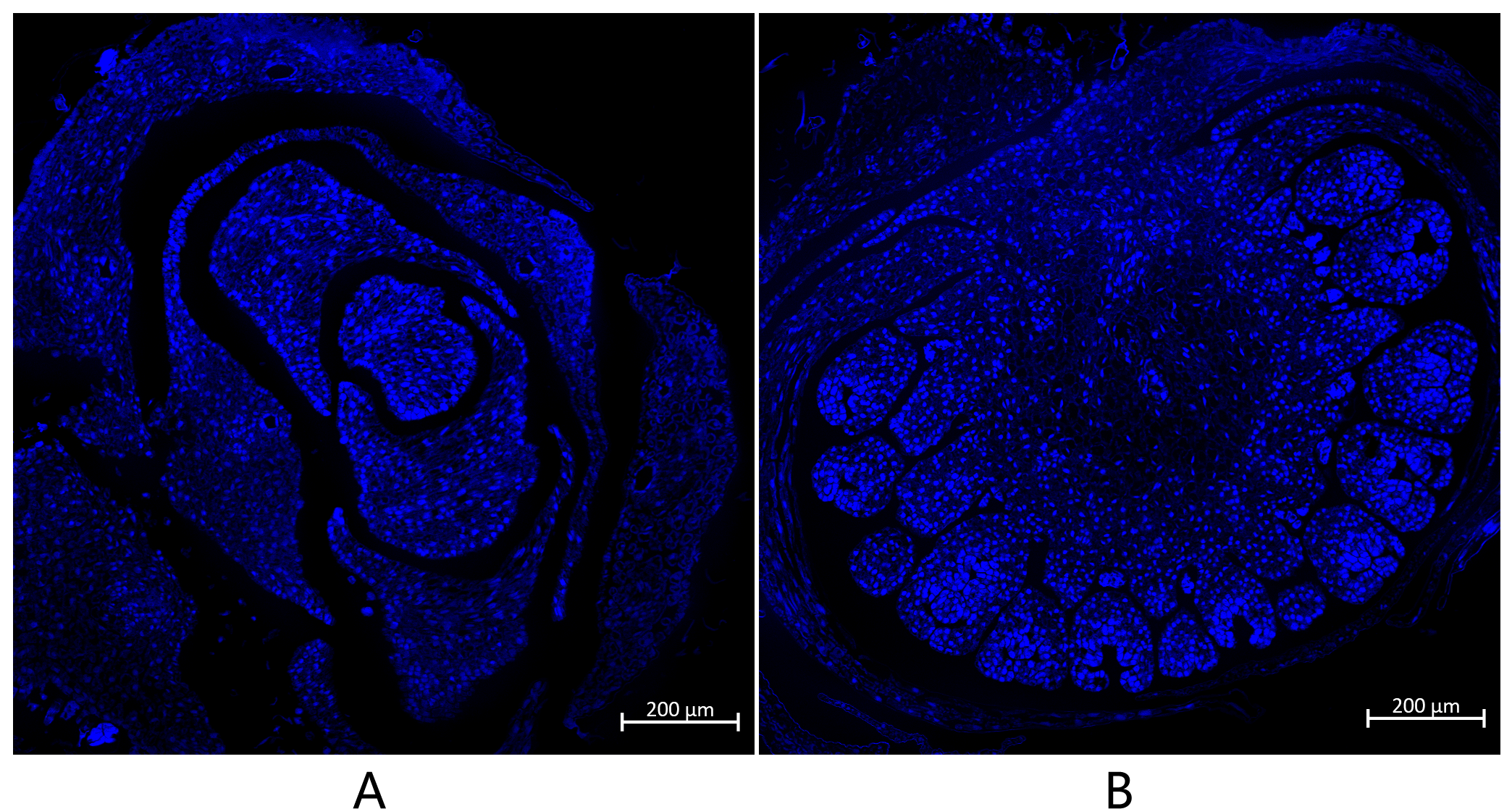

Supplement: Supplementary file 1 — Additional file 1. [file 12870_2021_2884_MOESM1_ESM.zip › Supplemental Fig. 3.tif]

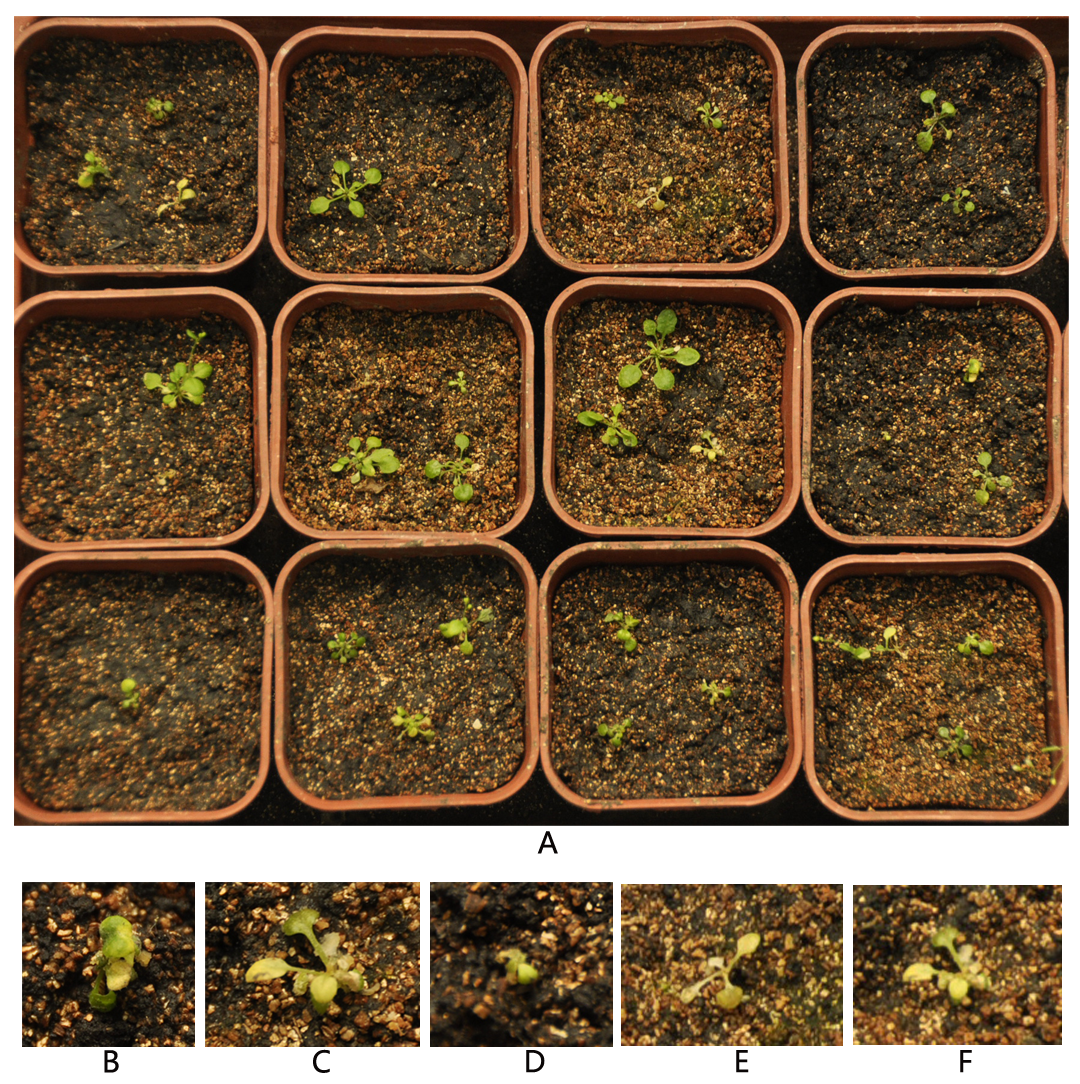

Supplement: Supplementary file 1 — Additional file 1. [file 12870_2021_2884_MOESM1_ESM.zip › Supplemental Fig. 4.tif]

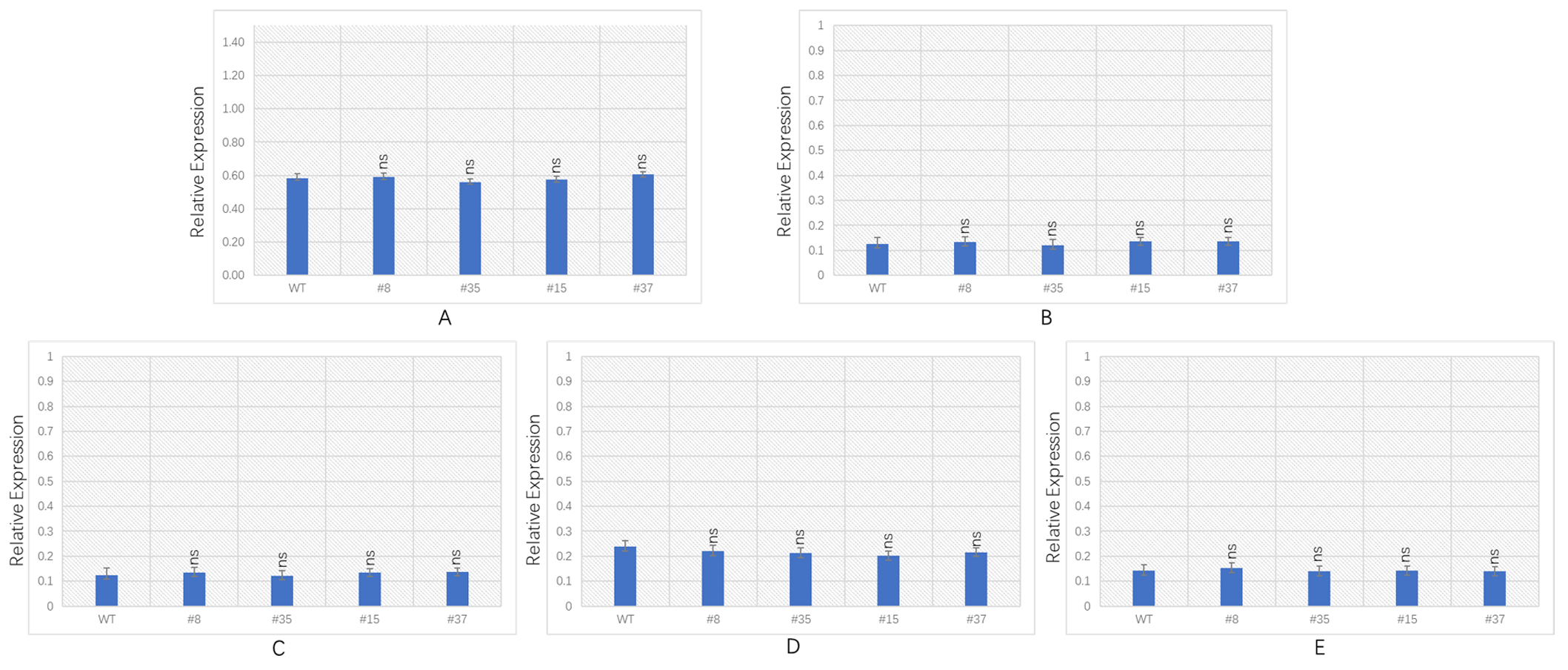

Supplement: Supplementary file 1 — Additional file 1. [file 12870_2021_2884_MOESM1_ESM.zip › Supplemental Fig. 5.tif]

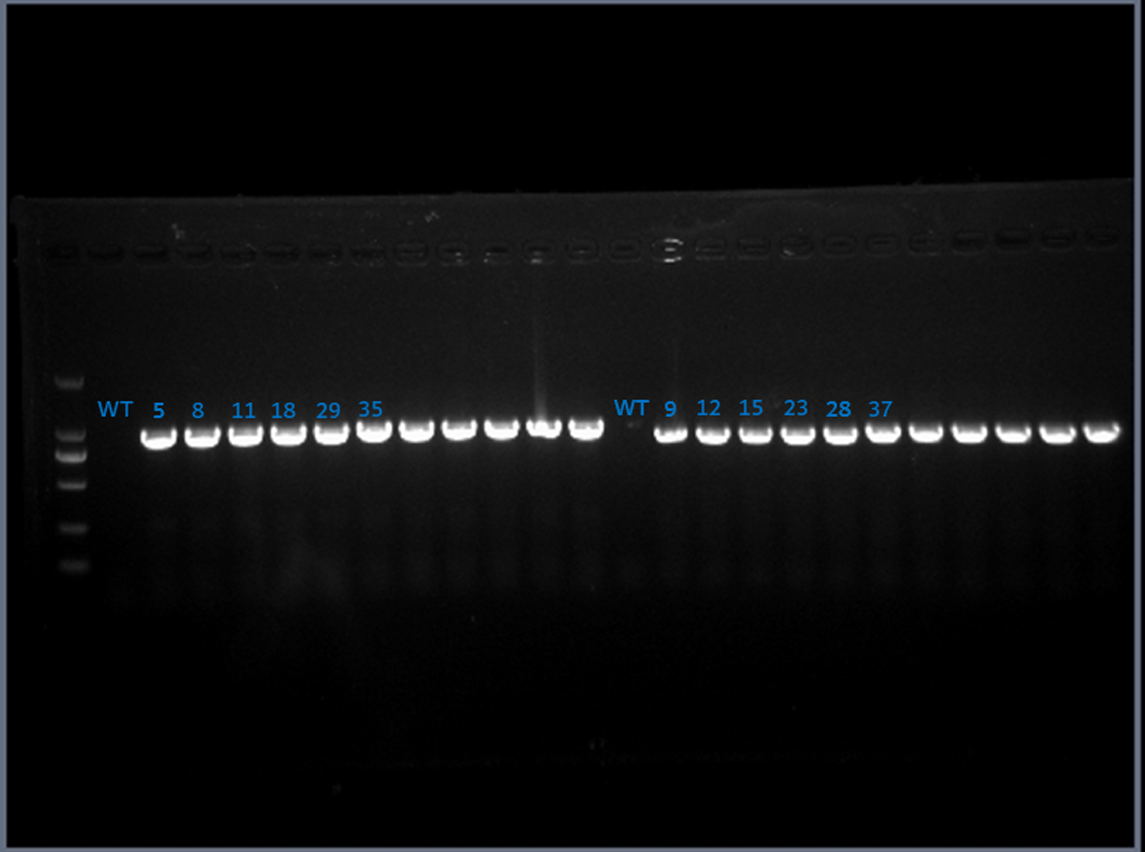

Supplement: Supplementary file 1 — Additional file 1. [file 12870_2021_2884_MOESM1_ESM.zip › Supplemental Fig. 6.tif]
